# Age and growth of one of the world’s largest carnivorous gastropods, the Florida Horse Conch, *Triplofusus giganteus* (Kiener, 1840), a target of unregulated, intense harvest

**DOI:** 10.1371/journal.pone.0265095

**Published:** 2022-04-06

**Authors:** Gregory S. Herbert, Stephen P. Geiger, Stephen G. Hesterberg, Nicole Seiden, Jaime A. Rogers, Ryan M. Harke, Martin Šala, Kaydee J. West, Ethan A. Goddard

**Affiliations:** 1 School of Geosciences, University of South Florida, Tampa, Florida, United States of America; 2 Florida Fish and Wildlife Conservation Commission, Fish and Wildlife Research Institute, St. Petersburg, Florida, United States of America; 3 Department of Integrative Biology, University of South Florida, Tampa, Florida, United States of America; 4 Department of Anthropology, University of South Florida, Tampa, Florida, United States of America; 5 Department of Analytical Chemistry, National Institute of Chemistry, Ljubljana, Slovenia; 6 University of South Florida College of Marine Science, St. Petersburg, Florida, United States of America; University of California, UNITED STATES

## Abstract

The Florida Horse Conch, *Triplofusus giganteus*, one of the largest marine gastropods in the world, has been intensely exploited by shell collectors, curio dealers, and commercial harvest for over a century and is now in decline. Effective management of horse conch populations requires better data on commercial and recreational harvest intensities but also on the species’ intrinsic capacity to recover. Here, we use stable oxygen and carbon isotope sclerochronology to investigate the horse conch’s life history, including its maximum life span, growth rates, age at first spawning, and number of lifetime spawning seasons. The largest two shells studied (460 and 475 mm linear shell length) grew for 13 and 11 years, respectively. Growth curves for these shells, extrapolated out to the length of the record size shell (606 mm linear shell length) predict a maximum age of just 16 years. Carbon isotopes and field photographs of spawning females suggest that females mature relatively late in life. However, the largest horse conchs remaining in the wild are also smaller and younger than those studied here. Thus, the largest females left in the wild could have few lifetime spawning events. High fecundity can buffer horse conchs from overfishing but only if females reach spawning age and reproductive-age females are protected. Our study highlights the usefulness of stable isotope sclerochronology for characterizing the life histories of molluscan species now too uncommon to study through traditional mark and recapture approaches.

## Introduction

The Florida Horse Conch, *Triplofusus giganteus* (Kiener, 1840) [1], which is found from North Carolina to Yucatán, Mexico, can reach shell lengths over 600 mm, making it one of the world’s largest carnivorous gastropods and one of the most highly sought after species by souvenir-hunting tourists, collectors, and commercial aquarium and curio shell dealers [2–4]. The horse conch was recognized as Florida’s official state seashell in 1969 due to its impressive size and use in the tourism industry as a symbol of the region’s abundant natural resources. However, decades of intense and mostly unregulated harvest, coastal development, and the species’ intrinsic vulnerability to extinction as a large-bodied, apex predator led the Florida Fish and Wildlife Conservation Commission to include *T*. *giganteus* on early lists of Species of Greatest Conservation Need [4–7].

Surveys of *T*. *giganteus* in Florida [2,6,8,9] post-date the start of intense harvest of this species by at least decades, making it difficult to know how far these populations are from baseline conditions [6]. However, early depletion of populations by shell collectors was reported by the 1960s [2,4]. More recent declines are documented in state-wide marine life landings of *T*. *giganteus*, which fell from a peak of 14,511 individuals in 1996, to 6,124 in 2000, 1,461 in 2015, and just 67 in 2020 [10], despite an increase in the number of fishing trips over roughly the same period [6]. Average densities of individual horse conchs surveyed along the west Florida coast from 2015 to 2017 were also lower than in Yucatán, Mexico, where the species is already considered to be overfished and in danger of local extinction [10,11]. Even in northwestern Florida, where horse conch densities are highest and populations appear to be stable [11], there is evidence for a substantial, post-1960s decrease in mean size [12]. Size decline is a common early warning signal of population collapse in overexploited fisheries [13].

In this study, stable isotope sclerochronology is used to estimate maximum life span, annual growth rates, timing of reproductive maturation, and lifetime spawning seasons for *T*. *giganteus*. Life history parameters for *T*. *giganteus* are currently unknown but critical for determining whether populations can replenish themselves at current rates of commercial and recreational harvest. Based on its large size, *T*. *giganteus* has been predicted to have a life span of many decades [11]. Given that an individual female can also produce up to 400 capsules, each with 70 hatching juveniles per capsule [14,15], high lifetime reproductive output may help buffer the species from harvest pressure [11]. However, some large, tropical marine gastropods, such as the Queen Conch *Aliger gigas* (Linnaeus, 1758), grow rapidly and reach sexual maturity at just over three years in age [16,17] and have an average maximum age of around a decade [18].

Stable isotope sclerochronology is well suited for characterizing the life histories of mollusks too uncommon and too mobile to study effectively with traditional mark-and-recapture methods. Oxygen isotope sclerochronology involves serial sampling and analysis of oxygen isotope values (δ^18^O) of sequentially-deposited shell layers. Seawater temperature typically exerts primary control on oxygen isotopes in molluscan carbonates formed in marine waters (inverse, linear correlation with shell δ^18^O) with secondary effects from seawater δ^18^O and negligible influence of physiology [19,20]. Thus, a typical δ^18^O profile from a gastropod shell records annual temperature cycles, which can also be used as an archive of information about the organism’s life history, such as age (number of annual cycles) and growth (distance per cycle). A previous isotope sclerochronology study on an adult male *T*. *giganteus* specimen from the Florida Keys found that its 340 mm length shell recorded just six annual δ^18^O cycles [21]. Given that the record size for the species is a shell 604.8 mm long measured from apex to siphonal canal tip and, like many organisms, had slow growth in the latter part of its life, the previous lifespan estimate of “many decades” [11] is not unreasonable.

Conservation-relevant information about reproduction can also be inferred from carbon stable isotope values (δ^13^C) in shells, which are influenced by dissolved inorganic carbon (DIC) in seawater and temperature but also the organism’s own respired CO_2_ [22]. Numerous studies have recorded ontogenetic shifts in δ^13^C values of mollusk shells (the “maturation jump”), which coincide with the onset of gametogenesis [23,24]. The exact mechanism by which this happens is unknown, but many gastropod species exhibit a sharp increase in body mass at the onset of maturity [25] that could indirectly affect δ^13^C_shell_ through changes in size- or growth rate-related physiology or diet, independent of gamete production. Here, we use trends in δ^13^C_shell_ in *T*. *giganteus* shells and additional supporting evidence from shell lengths of photographed spawning females in the wild to propose a hypothesis for age at first reproductive maturation.

Finally, isotope sclerochronology profiles are used to test whether morphological features of *T*. *giganteus* opercula are formed at regular seasonal intervals as they do in some other gastropod species [26–28]. If so, visual counts of these features could be used for rapid, non-lethal, field assessments of age.

## Materials and methods

### Sample selection

**Fig 1:**
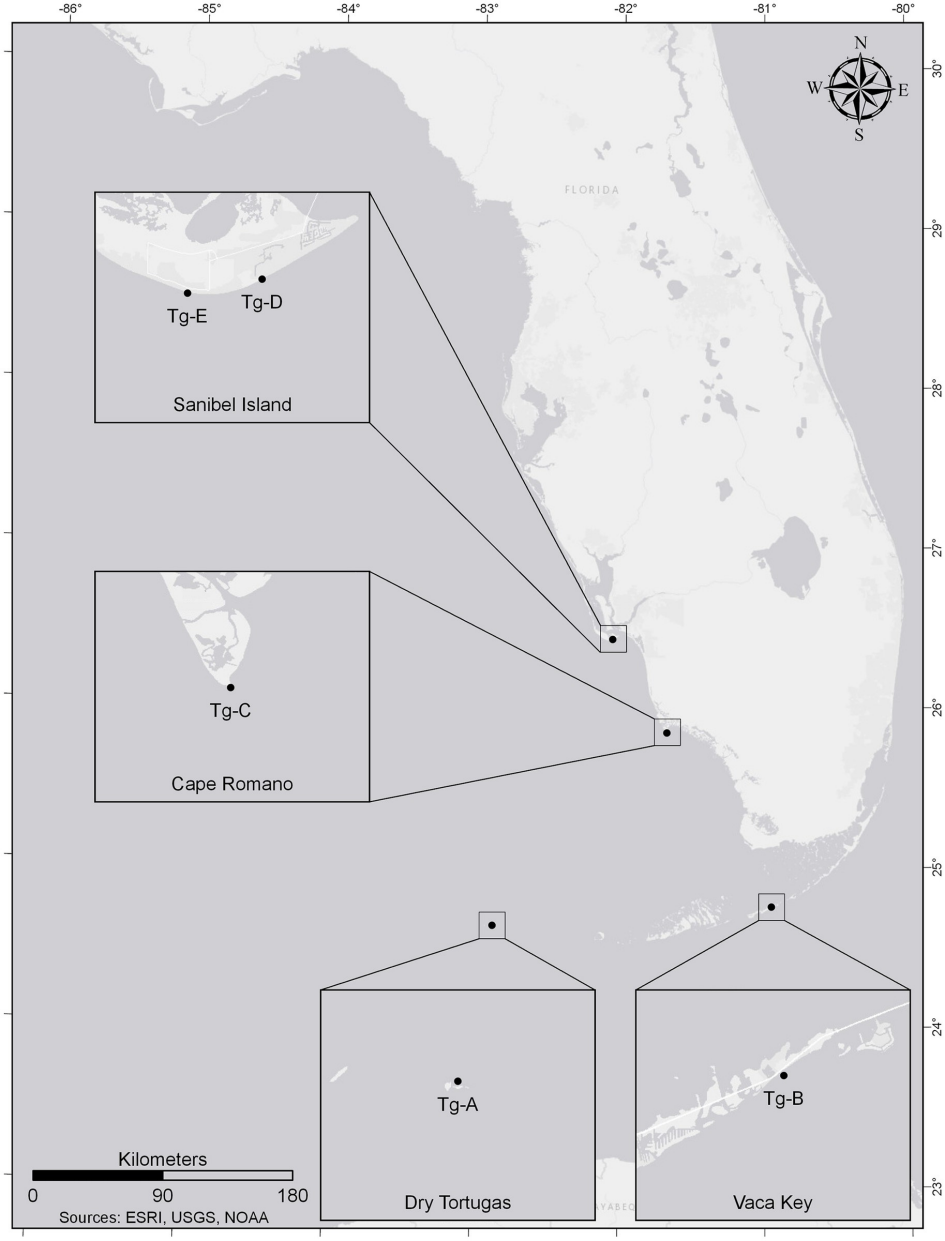
Map of sampling localities. Map of Florida, USA showing collection localities for shells of the Florida Horse Conch, *Triplofusus giganteus*, used for stable isotope sclerochronology.

Isotope sclerochronology was performed on five specimens of *T*. *giganteus* (Tg), with a focus on populations from Florida (Fig 1), the center of the species’ range and region of the most intense, unregulated harvest. These include four shells in the Bailey-Matthews National Shell Museum (BMSM) collections: Tg-A (BMSM 119422) from the Dry Tortugas, Tg-B (BMSM 119423) from Vaca Key, and juvenile specimens Tg-D and Tg-E (both BMSM 25347) from Sanibel Island. Specimen Tg-C was live-collected off Cape Romano in 2013 and is housed in the collections of one of the authors (G.S.H.) at the University of South Florida.

Tg-A (linear shell length = 460 mm) and Tg-B (linear shell length = 475 mm) were selected because they are among the largest shells known for the species in Florida outside of the record length shell and allow estimation of the true maximum age within Florida populations. The record length shell (604.8 mm), on display at the BMSM, was not available for destructive isotope sampling, although growth curves derived from Tg-A and Tg-B were extrapolated to estimate its age and the maximum age of the species (below). Tg-C (linear shell length = 311 mm), an intermediate-size shell for the species, was sampled first at coarser resolution to supplement information in Strauss et al. [21] on the sample spacing required to resolve life histories of the largest shells. The two juvenile shells, Tg-D (linear shell length = 36 mm) and Tg-E (linear shell length = 37 mm), were analyzed isotopically to whether any annual cycles occur in the first several shell whorls, which are missing in the largest shells due to erosion.

### Stable isotope geochemistry

Shells were prepared for stable isotope sclerochronology by cleaning with a 3% hypochlorite solution to remove organics, scrubbing with a soft-bristle brush or pick to remove any remnant parts of the periostracum, washing with DI water, and air drying. At each point, ~100 μg of aragonite powder was micromilled in a <1 mm deep track using a hand-held drill with a 0.5 mm bit from the outermost microstructural layer, parallel to growth lines, and starting at the suture. The first sample on each shell was positioned at the earliest preserved point on the spire as close to the protoconch-teleoconch boundary as possible, and additional samples were added sequentially at tight intervals following the spiral growth to the shell lip.

Following Schöne et al. [29], we used “adaptive sampling” to determine the sample spacing needed to resolve annual cycles in the isotope profiles. This method, which is most efficient for large shells, applies coarser resolution (~7 mm) for fast-growing juvenile whorls and tighter spacing (~1 mm) near the terminal adult lip, when growth rates are expected to decline. In the final profiles, adaptive sampling resulted in 158 total samples and an average of 12 samples/year for Tg-A, 169 total samples and an average of 15 samples/year for Tg-B, and 56 total samples and an average of 6 samples/year for Tg-C. Similar sampling methods by Strauss et al. [21] for a single *T*. *giganteus* shell were adequate to resolve annual temperature cycles. We also sampled two juvenile shells (Tg-D and Tg-E) at even 2.5 mm intervals along the growth axis for a total of 18 samples each.

Samples for shells Tg-A, Tg-B, Tg-D, and Tg-E were analyzed by dissolving samples in 100% H_3_PO_4_ at 50° C for 90–180 min with a ThermoFisher MAT253 stable isotope ratio mass spectrometer coupled to a GasBench-II peripheral in continuous-flow mode located at the University of South Florida College of Marine Science Marine Environmental Chemistry Lab. Secondary reference materials (TSF-1 δ^13^C = 1.95 ± 0.05 ‰, δ^18^O = -2.20 ± 0.06 ‰; Borba δ^13^C = 2.87 ± 0.05 ‰, δ^18^O = -6.15 ± 0.09 ‰; LECO-carb δ^13^C = -15.45 ± 0.16 ‰, δ^18^O = -20.68 ± 0.16 ‰, all calibrated with NBS19, NBS18 and LSVEC certified reference materials) were used to normalize measurements to the VPDB scale. Measurement uncertainty, expressed as ± 1 standard deviation of n = 81 measurements of the TSF-1 laboratory reference material, was ± 0.01 ‰ for both δ^13^C and δ^18^O. Samples for Tg-C were analyzed by dissolving samples in 104% H_3_PO_4_ at 50° C for 2 h, and analyzing the resulting gas using a ThermoFisher Scientific Delta V Isotope Ratio Mass Spectrometer and GasBench II in the School of Geosciences’ Stable Isotope Laboratory at USF. Secondary reference materials (TSF-1 δ^13^C = 1.95 ± 0.05 ‰, δ^18^O = -2.20 ± 0.06 ‰; UCDSM92 δ^13^C = 2.06 ± 0.14 ‰, δ^18^O = -1.91 ± 0.45, all calibrated with NBS18 certified reference materials) were used to normalize measurements to the VPDB scale. Measurement uncertainty, expressed as ± 1 standard deviation of n = 42 measurements of the TSF-1 laboratory reference material, was ± 0.06 ‰ and ± 0.10 ‰ for δ^13^C and δ^18^O, respectively. Isotope ratio values are expressed in δ ‰ (= [(R_sample_−R_standard_)/R_standard_].

Following Strauss et al. [21], annual cycles in the isotope profiles are defined as sinusoidal variation in δ^18^O shell values of ~2 ‰ amplitude, with both years and spiral distance records measured beginning at the earliest sampled point at the shell apex. However, because many mollusks switch from rapid to slow growth late in ontogeny [30,31], the amplitude of annual cycles was expected to decrease with age as a result of decreased sampling resolution.

### Laser ablation-inductively coupled plasma-mass spectrometry

To test whether there might be additional years of very slow growth near the shell lips of the largest, oldest shells, high resolution LA-ICP-MS of Mg/Ca and Sr/Ca ratios was performed along a 14 cm long section of shell near the lip from Tg-A and a 7 cm long section near the shell lip from Tg-B. Like δ^18^O in molluscan shells, Mg/Ca and Sr/Ca ratios can vary seasonally in mollusks [32–34], but the effects can vary strongly between species [35–37]. Rectangular thin sections were removed from Tg-A and Tg-B lips with a dremel cutting tool, embedded in epoxy, mounted on glass slides, and sanded flat.

Five replicate laser line transects were taken at equidistant intervals along each shell section using a laser ablation system (193 nm ArF* excimer; Analyte G2 Teledyne Photon Machines Inc., Bozeman, MT) that was equipped with a standard active two-volume ablation cell (HelEx II), including the Aerosol Rapid Introduction System (ARIS, Teledyne CETAC Technologies) for fast aerosol washout. The laser ablation unit was coupled to a quadrupole ICP-MS instrument (Agilent 7900x, Agilent Technologies, Santa Clara, CA). Ablation parameters were as follows: laser energy density, 3.0 J cm−2; repetition rate, 275 Hz; beam diameter, 20 μm (square mask); dosage 11 and total acquisition time for ICP-MS acquisition was 40 ms (with corresponding dwell times for specific nuclides: ^24^Mg, 5 ms; ^43^Ca, 5 ms; and ^88^Sr, 5 ms). Other parameters were based on model predictions for fastest possible mapping times, avoidance of aliasing, minimal blur and maximal S/N ratios [38]. Data processing and image analysis were performed using the software packages HDIP (Teledyne Photon Machines Inc., Bozeman, MT) and ImageJ.

Elemental count data were converted to mmol/mol by a two-point calibration using the JCt-1 standard (Geological Survey of Japan) [39–41]. Following Marali et al. [42], mmol/mol ratios exceeding 31-point running averages by 5σ were deemed outliers and removed. Intra-specimen reproducibility was confirmed by averaging every 50 consecutive data points of each line and comparing lines using Pearson Correlation in SPSS (version 26). Because trace metal variation was synchronous across lines, the five lines of each section were averaged to create a single final line for each trace element and then aligned with δ^18^O profiles using known distance measurements of the section.

### Opercular ring counts of age

External striae and interior adventitious layers of the operculum were counted for Tg-A and Tg-C, the only two of our shells for which opercula were available. Annual striae are defined following Vasconcelos et al.’s [43] criteria: (1) continuity around the operculum to the edge, and (2) an obvious change in surface topography. Adventitious layers (layers added on the interior surface of the operculum) are visible as dark and light bands at the abapical, pointed end of the operculum’s interior surface.

### Age and growth curve models

To construct size-at-age relationships, oxygen and carbon isotope values were plotted against spiral length, which is the distance in mm along the spiral axis of growth. Spiral length was measured by wrapping string tightly around the suture towards from the apex to the shell lip and marking the positions of each milled isotope sample on the string. The relationship between spiral length and shell length (the straight line from apex to canal) is linear as determined by measurements of 72 shells between 6.0 and 604.8 mm in shell length in the Bailey Matthews National Shell Museum:

$$Spiral\;length\;(mm)=[1.99\;*\;shell\;length\;(mm)]\;+\;0.45\;(R^{2}=0.99,\;p\;<\;0.001)$$ (1)

Growth was modeled with spiral-length-at-age data using linear, von Bertalanffy, logistic, and Gompertz growth curves in PAST 4.03. The Gompertz curve, which provided the best fit overall as measured by AIC, was used to model all growth curves. Spiral growth data from a specimen (here designated Tg-S) from the Florida Keys published previously [21] were included in this analysis. Growth curves were extended out to the spiral length of the record size shell to model the maximum age-at-size for the species. To determine the “true” size of the record shell, however, the spiral length of the missing uppermost whorls, which are eroded in all larger, older *T*. *giganteus*, was estimated and added to the measured spiral length. To do this, the diameter of the record shell’s earliest preserved whorl was converted to its missing spiral length using the empirical relationship between maximum whorl diameter and total spiral length generated from 41 complete juvenile shells (lots BMSM 25353, BMSM 25347) with shell lengths between 8.1 and 51.2 mm. This relationship is defined as:

$$Spiral\;growth\;distance\;(mm)=[3.539\;*\;whorl\;diameter\;at\;lip\;(mm)]\;-\;0.053\;(R^{2}=0.94,\;p\;<\;0.001)$$ (2)

### Ethics statement

Specimen Tg-C was collected under Florida Fish and Wildlife Research Institute special activity license SAL-11-0901C-SR to the lead author (G.S.H.).

## Results

### Oxygen isotope and trace element sclerochronology

**Fig 2:**
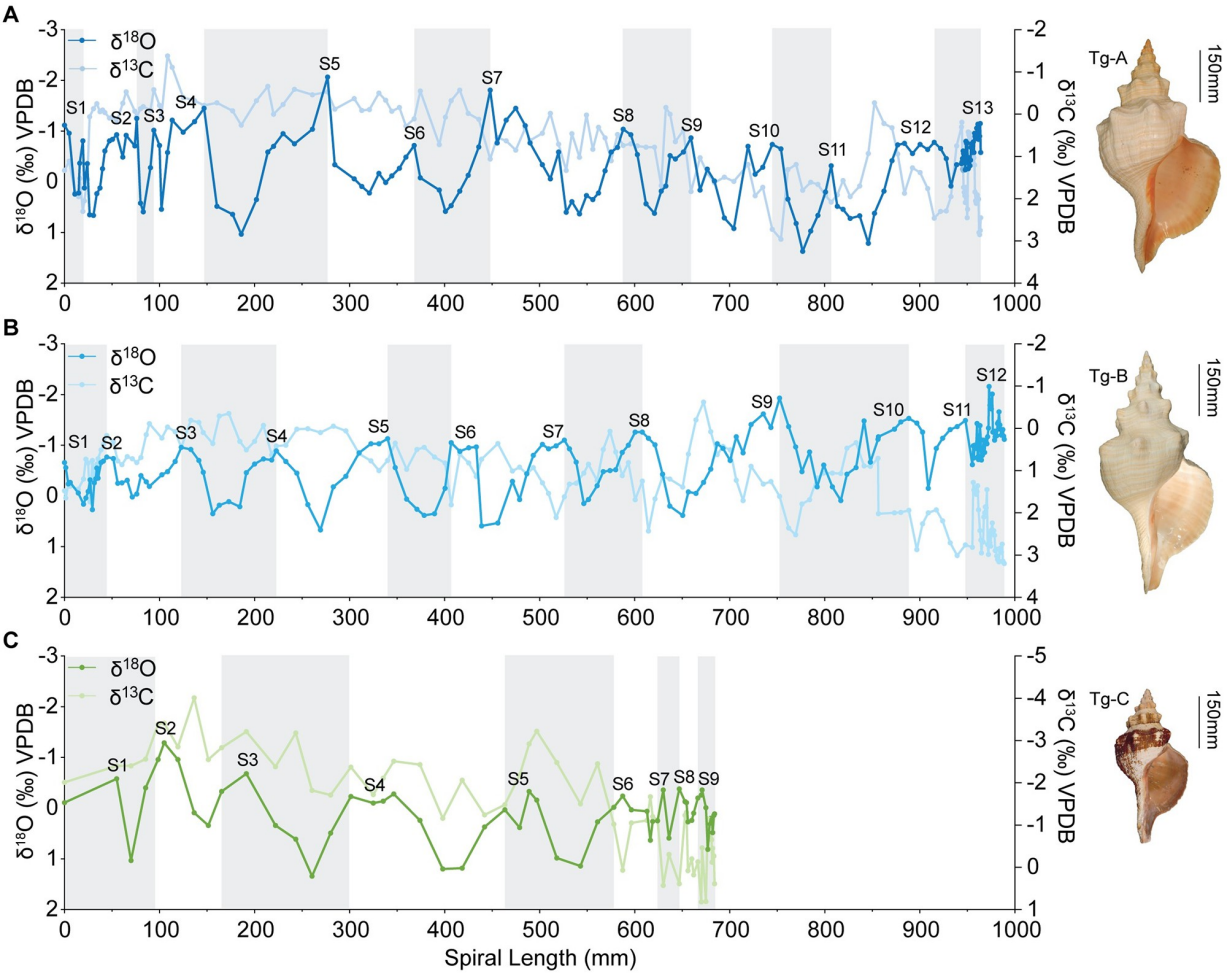
Stable oxygen and carbon isotope profiles. Oxygen (darker lines) and carbon (lighter lines) stable isotope sclerochronology profiles for *Triplofusus giganteus* shells. A. Specimen Tg-A (BMSM 119422) from the Dry Tortugas, Florida Keys. B. Specimen Tg-B (BMSM 119423) from Vaca Key, Florida Keys. C. Specimen Tg-C from Cape Romano, Florida. Positions of summers (S) are indicated at cycle peaks. Years are shown as alternating grey and white bands. Line colors correspond to growth curve colors in Fig 4.

Oxygen isotope profiles for all three shells show annual cycles defined by lower δ^18^O values (higher temperatures) and higher δ^18^O values (lower temperatures) (Fig 2). Maximum amplitudes for individual annual cycles are 3.0 ‰ for Tg-A, 2.3 ‰ for Tg-B, and 2.3 ‰ for Tg-C. In fully marine waters (> 35 PSU), these amplitudes correspond to a seasonal range of roughly 9 to 12°C, which is comparable to or near the mean annual ocean temperature ranges for the Florida Keys (10°C) and Cape Romano (15°C) collection areas (https://www.ndbc.noaa.gov/). Lower amplitude, shorter-period cycles are observed in early and late ontogeny of each profile. Cool season temperatures were truncated and growth rates were reduced in the last year in Tg-A and Tg-B and last four years of Tg-C.

Ages of specimens, determined as the number of δ^18^O annual cycles, are 13 y for Tg-A, 11 y for Tg-B, and 9 y for Tg-C (Fig 2). Isotope profiles of two complete juvenile shells (Tg-D and Tg-E), both slightly larger than the juvenile whorls missing from larger, older shells, each have δ^18^O amplitudes around 0.5 ‰ (S2 Fig). This range is several times smaller than the amplitudes of the largest annual cycles seen in larger shells and consistent with a total growth duration of months.

Mg/Ca and Sr/Ca profiles exhibited variation that was largely uncorrelated with δ^18^O values for both Tg-A and Tg-B sections (S1 Fig). This is especially apparent in the longer section of Tg-A. Here, the amplitudes of Mg/Ca and Sr/Ca exhibit a constant range of variation (around 0.8 to 0.6 mmol/mol, respectively), whereas the amplitude of δ^18^O decreases from roughly 2 ‰ between 830 and 930 mm to around 1 ‰ for the rest of the profile. Neither trace element profile can be interpreted as revealing extra years of growth left unresolved by oxygen isotopes.

### Opercular ring counts of age

**Fig 3:**
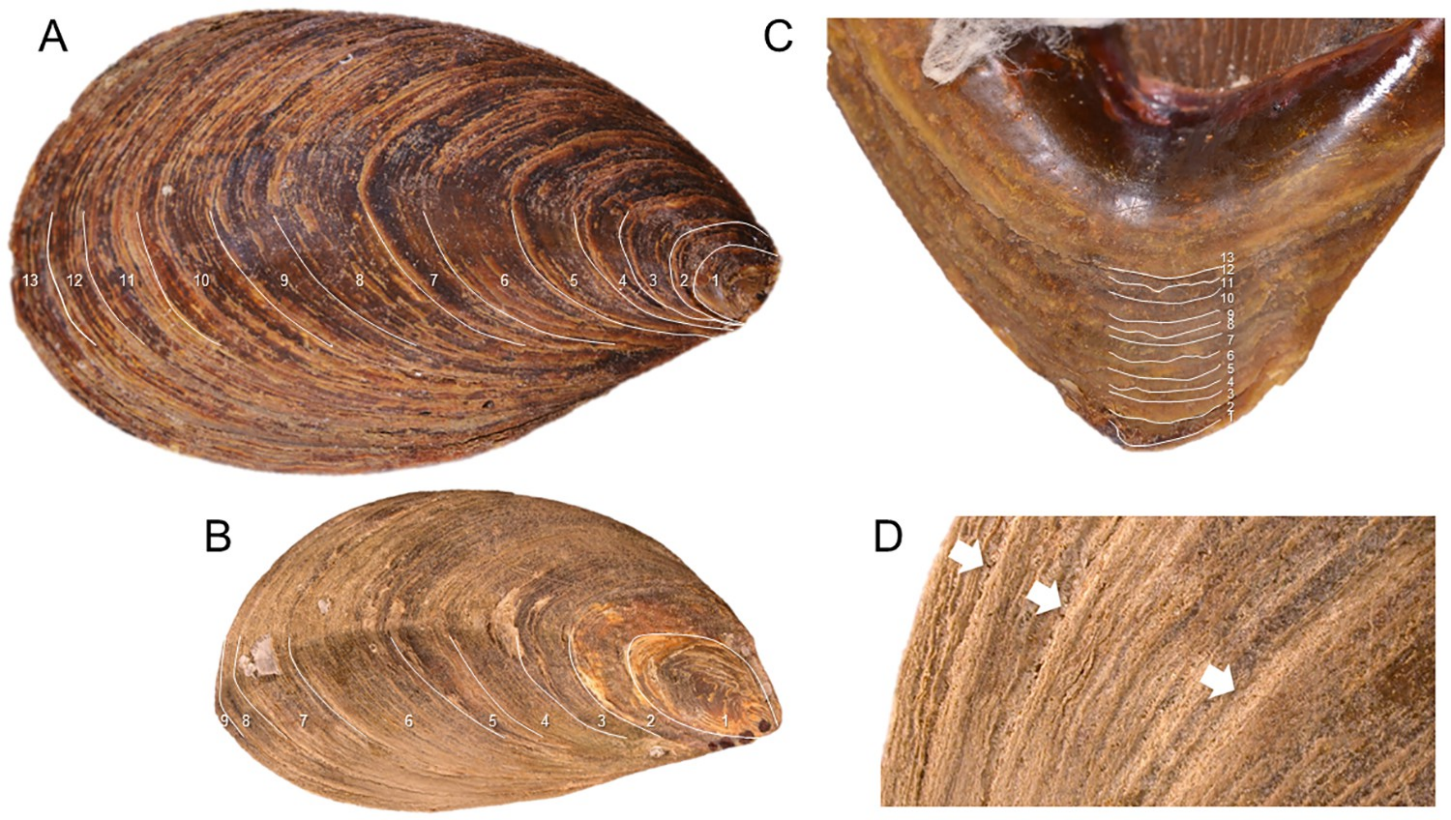
Counts of opercular striae and adventitious layers. Opercula showing annual striae (A, B, D) on external surface and interior adventitious layers (E). A, C, D. Specimen Tg-A (BMSM 119422) from the Dry Tortugas, Florida Keys. B. Specimen Tg-C from Cape Romano, Florida. White lines indicate annual striae and adventitious layers. Arrows mark sharp ledges of annual striae on the external surface.

External striae and internal adventitious layer counts matched one another for Tg-A (N = 13) and Tg-C (N = 9) (Fig 3) and also matched oxygen isotope-based estimates of specimen age.

### Growth curve and age models

**Fig 4:**
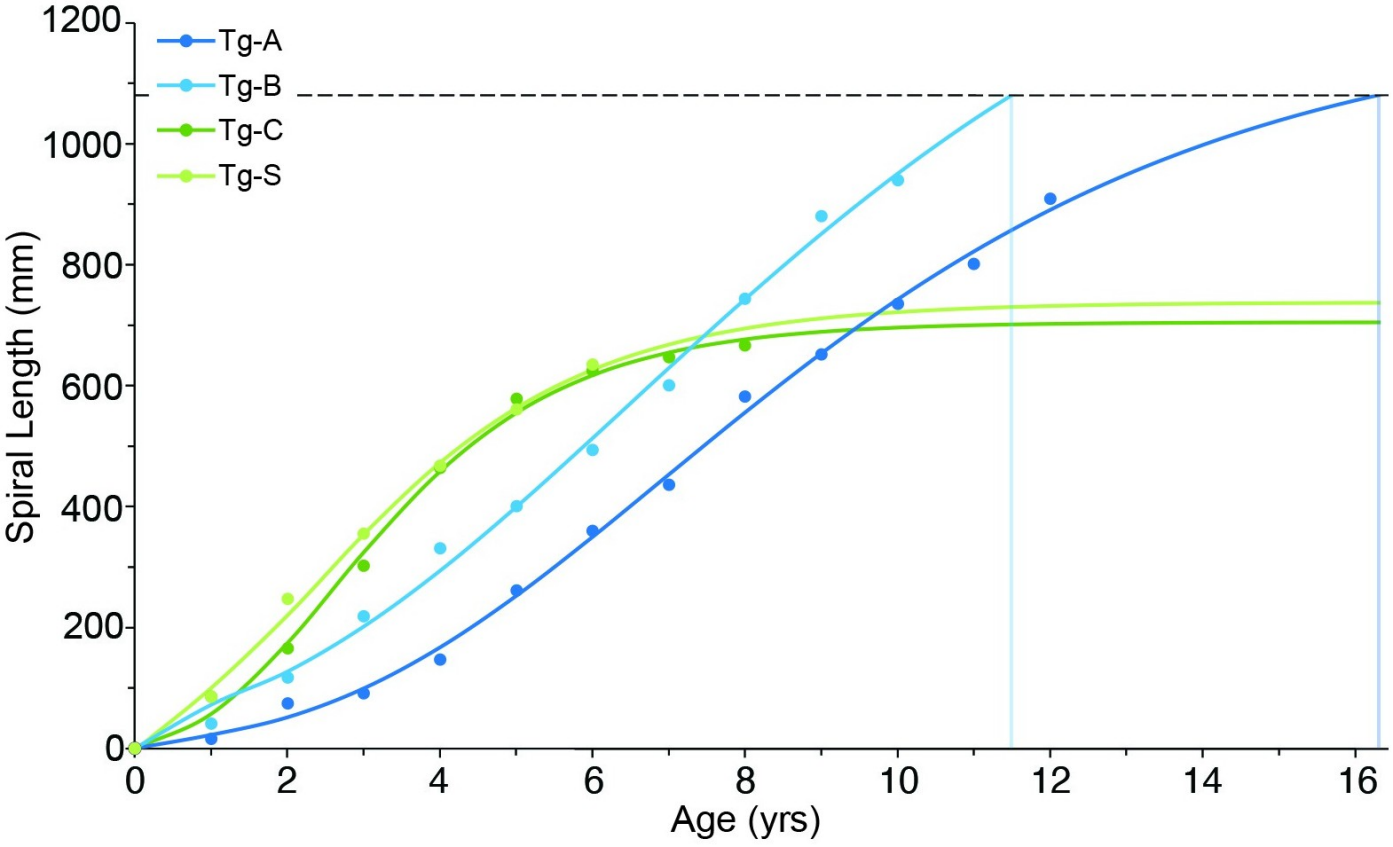
*Triplofusus giganteus* growth curves. Growth curves for Tg-A, Tg-B, Tg-C (this study), and a male specimen Tg-S [from Strauss et al., 21] modeled with spiral-length-at-age data from oxygen isotope profiles. The horizontal dotted line represents the measured spiral length of the record size shell. Vertical lines are placed at the intersection of growth curves with this horizontal line to show how long it would take for observed growth patterns in *T*. *giganteus* to produce the record shell size.

Cumulative growth curves for Tg-A, Tg-B, Tg-C, and Tg-S followed one of two sigmoidal patterns: (1) for Tg-C and Tg-S, rapid growth that levels off around year 6 between 600–700 mm spiral length (300–350 mm linear shell length), and (2) for Tg-A and Tg-B, slower growth that does not level off with time (Fig 4). To derive age estimates of the record size shell, growth curves were extrapolated out to the spiral length of the record-size shell. Because the upper-most whorls of the record shell are eroded, the diameter of the record shell’s first preserved apical whorl (5.53 mm) was first used to estimate the spiral length missing from its apex using Eq 2. The estimated missing spiral length of 19.5 mm was added to the measured spiral length of 1079.5 mm to bring the total corrected spiral length to 1099 mm. Extrapolating all four cumulative growth curves, shells with the more rapid growth pattern (Tg-C, Tg-S) level off early without reaching the record length of 1099 mm. Shells with the slower growth pattern reach 1099 mm at around 11 y (Tg-A model) and 16 y (Tg-B model), respectively (Fig 4).

### Carbon isotope sclerochronology

All three profiles exhibit the same four temporal phases in δ^13^C: (1) higher isotope values within the first year, (2) a rapid shift to lower δ^13^C values over the next several years, (3) a gradual, long-term trend of 3 to 4 ‰ towards the highest δ^13^C values in the profile, and (4) a relatively short plateau at the highest values (Fig 2). Specimen Tg-B is the only shell to show occasional negative covariation with δ^18^O (from the 4^th^ to 8^th^ summers) (Fig 2).

## Discussion

### Age determination

Oxygen isotope profiles reveal 13 full years of growth in Tg-A, 11 years in Tg-B, and 9 years in Tg-C, all exceeding Strauss et al.’s [21] previous isotope sclerochronology-based age of 6 years measured for *T*. *giganteus*. Because several of the uppermost whorls of all three shells were missing due to erosion, the ages determined using the isotope sclerochronology method could be underestimates. However, δ^18^O_shell_ profiles were also constructed for two, complete, juvenile shells (Tg-D, Tg-E) that would have been slightly larger than the missing apex whorls of Tg-A, Tg-B, and Tg-C. Both juvenile shell profiles exhibit low amplitude (sub-seasonal) δ^18^O variation and represent a period of growth of a few months at most. Thus, the loss of earliest whorls due to erosion on the larger shells has not resulted in missing years from our sclerochronology-based age interpretations.

Sclerochronology-based ages might also be underestimated if shell growth stopped or slowed below the limits of sampling resolution at any point during the animal’s life. Growth slow-down in late ontogeny was observed in Tg-C as a sharp reduction in the amplitude and growth distance of cycles, but these cycles and those of Tg-A and Tg-B were resolved by increasing sampling resolution at the shell lip. High resolution (20 μm) sampling with laser ablation (Mg/Ca and Sr/Ca) also did not reveal evidence for additional annual cycles not already captured by isotope data. Agreement between isotope-based age reconstructions for *T*. *giganteus* with year counts from external opercular striae and internal opercular adventitious layers further supports these age estimates. This consensus also means that the cheaper, faster, non-lethal method of counting opercular striae can be used reliably for age assessments of *T*. *giganteus* in field surveys and historical museum collections.

### Maximum lifespan

Sigmoidal growth curves best model shell growth in *T*. *giganteus*, as they do in many other mollusk species [44,45]. However, among the four individuals considered in this study are two distinct developmental patterns. The two smaller shells, Tg-C and Tg-S, both have a more rapid exponential phase and shorter logarithmic phase compared to the much larger Tg-A and Tg-B. Given that Tg-S is a male [21] and that female neogastropods tend to reach much larger adult sizes than males [46], the growth patterns observed in the smaller vs. larger shells are hypothesized to be male-female sex-based differences.

The rapid growth pattern of the two smaller shells occurred in one individual from an intertidal, estuarine habitat (Tg-C) and a male specimen from a subtidal, oligotrophic reef (Tg-S). Conversely, both the rapid and slow growth patterns were observed in individuals from a subtidal, oligotrophic reef environment in the Florida Keys (Tg-A, Tg-B, Tg-S). Thus, while growth is undoubtedly influenced by environmental controls, we propose that growth curve patterns in this species are controlled more strongly by an intrinsic biological factor, such as the snail’s sex.

Interestingly, the growth curves of the two smaller shells level off before reaching the spiral growth distance of the record-size shell. In contrast, growth curves of the larger shells are nearly linear and reach the record shell size at 11 (Tg-B) and 16 years of age (Tg-A). This maximum lifespan estimate for *T*. *giganteus* is significantly shorter than a previous prediction of many decades based on its size alone [11]. This has important implications for management of the species as described below.

### Timing of reproductive maturation and lifetime spawning events

Higher δ^13^C values (3 to -1 ‰) in shells from the more oligotrophic Florida Keys (Tg-A, Tg-B, Tg-S) and lower values (1 to -4 ‰) in the shell (Tg-C) from Cape Romano, which is directly adjacent to the Florida Everglades and heavily influenced by mangrove and wetland productivity, are consistent with expected DIC trends [47]. However, despite these location differences, each of the four profiles considered here (including Tg-S) exhibit similar ontogenetic trends towards higher δ^13^C values.

Strauss et al. [21] interpreted the long-term trend towards higher δ^13^C values in Tg-S as tracking migration of the snail into waters with isotopically-heavier dissolved inorganic carbon (DIC). However, the magnitude of the ontogenetic trend within shells is comparable to the difference in mean values of shells from the oligotrophic Keys reef versus Everglades-influenced-estuary [47], which is not a realistic distance for Tg-S, a six-year-old snail, to traverse. Additionally, corresponding δ^18^O values (a temperature indicator) in the four shells considered here do not exhibit consistent trends as expected if they were tracking the same environmental gradient as δ^13^C during a migration. Although δ^13^C values increase over time in all four shells, δ^18^O values increase in Tg-A, show no trend in Tg-C, and decrease in Tg-B and Tg-S [21].

Large ontogenetic shifts in δ^13^C_shell_ sometimes coincide with the general timing of gametogenesis in large-bodied mollusks, including non-migratory bivalves [23,24], although the causal mechanisms for this correspondence are unknown, and the trends may be sudden or gradual and shift towards lower or higher δ^13^C values depending on the species. In *T*. *giganteus*, the shift is gradual and trends towards higher δ^13^C values. In the two largest and presumably female *T*. *giganteus* shells, Tg-A and Tg-B, the trend begins around 250 mm spiral length (~125 mm linear shell length) and continues until a plateau at around 750 mm spiral length in Tg-A (~ 375 mm linear shell length) and 800 mm spiral length in Tg-B (~ 400 linear shell length). There is no sudden maturation jump. The Queen Conch *Aliger gigas* (Linnaeus, 1758), however, also has a protracted δ^13^C trend, with a known age of maturation coinciding approximately with the first jumps in δ^13^C amplitude [24]. If we assume the same to be true for *T*. *giganteus*, then female maturation was reached by a spiral shell length of 400 to 500 mm (~ 200 to 250 mm linear shell length), and an age of six (Tg-B) or seven (Tg-A) years. Had they lived as long as the maximum estimated age of the record size shell, these animals may have had up to nine or ten years of reproductive maturity.

If Wefer and Berger [24] are correct in inferring that each large δ^13^C jump corresponds to an individual spawning event, then it may also be possible to track the number of successful spawning events for an individual female from its isotope profile and not just the number of years of maturity. For example in the profile for Tg-B, there are regular jumps in δ^13^C during cooler months for most of the profile after Year 5, but just one jump per year. In the final year of growth, there are three or four δ^13^C jumps of gradually declining amplitude, which could indicate multiple spawning events of decreasing egg production. In contrast, Tg-A has smaller cool season δ^13^C jumps than Tg-B and some years with no δ^13^C jumps, which may indicate no spawning occurred. If this hypothesized relationship between δ^13^C jumps and spawning is confirmed for this species, δ^13^C_shell_ data could provide highly detailed data on year-to-year variation in spawning frequency at the level of individuals and localities that could not be obtained by any other means.

Ground truthing our hypothesized age-at-first-spawning in *T*. *giganteus* is inherently difficult due to the rarity of large, reproductive age females in the wild and the infrequency of spawning events. Despite a nearly decade-long survey of horse conchs along the Florida west coast, Geiger et al. [11] reported observing just one spawning female. However, a giant female horse conch spawning an egg mass nearly as large as itself is a visually stunning sight, and nearly a dozen spawning events have been documented by SCUBA divers and tourists in photographs and videos uploaded to social media. All of the spawning females documented this way are large (e.g., https://www.flickr.com/photos/myfwc/33173027940/in/album-72157678208717314/; https://www.youtube.com/watch?v=pmkLVn3YOKc, accessed 9/26/21). Using an egg capsule width of 2 cm [48] as a standard scale, the smallest egg-laying female photographed (https://www.jaxshells.org/pleggs.htm, accessed 9/26/21) is estimated to have been around 260 to 280 mm linear shell length, which is close to the linear shell length of 200 to 250 mm at first spawning predicted by carbon isotopes. All together, evidence on age of maturation from carbon isotopes and social media images and rarity horse conch spawning events documented in ecological survey data support the hypothesis that female maturation is delayed in *T*. *giganteus*, and that females have few spawning years in a lifetime. Our hypothesis of delayed maturation could most efficiently be tested with cool-season field surveys targeting females specifically within 200 to 250 mm size window for signs of gonadal development and spawning.

### Management implications

Maximum shell lengths of *T*. *giganteus* recorded in the most recent ecological surveys along the west Florida coast are not much larger than the predicted minimum size at first spawning. For example, the largest recorded linear shell length in a recent Tampa Bay survey was 341 mm (a spawning female) [11], which corresponds to a spiral length of ~ 679 mm and an age between seven and nine years using growth curves for the larger Tg-A and Tg-B shells. Thus, assuming an earliest female maturation age of six or seven years, the largest females encountered in Tampa Bay may have, at most, one to three spawning years over a lifetime. In northwest Florida, Kuhlmann [9] reported 385 mm as the largest linear shell length (~ 770 mm spiral length) observed in a survey of *T*. *giganteus* from St. Joseph Bay, while Paine [2] and Rogers and Kimbro [12] both found maximum sizes at Bay Mouth Bar around 400 mm linear shell length (~ 800 mm spiral length). Using the same earliest maturation age and growth curves, we predict that females this size have three to five years of spawning per lifetime.

Geiger et al. [11] argued that high numbers of hatching offspring per spawn could help *T*. *giganteus* sustain itself from harvest pressure, but this works only if animals reach maturity and spawning-size females are protected. Our findings suggest instead that delayed maturation and short average lifespans could combine to create a life history-trap in *T*. *giganteus* that increases risk of collapse for populations, particularly for those that are intensely fished. Fisheries limits that allow removal of the largest members of the population not only reduce the number of spawning years but disproportionately impact the most productive females. One management approach could be to focus on slot limits: minimum sizes to allow at least one spawning event and maximum sizes to protect the most productive breeding individuals [5–7]. Assessing whether an unprotected species such as *T*. *giganteus* would benefit from these protections, however, requires formal assessment based on additional information on its abundance, range, and demographic structure. The wide distribution at low densities of *T*. *giganteus* buffers the species from future commercial-scale exploitation, but at times they have been spotted in aggregations at south Florida beaches (https://www.facebook.com/shellmuseum/photos/a.149245819700/10153989703919701, accessed 9/2/2021), and the limited landings data suggest unpredictable patches of higher density. If these events are related to spawning, which seems likely, then protections for specific locales or seasons might be justified. The addition of Marine Protected Areas (MPAs) and protections of spawning areas and seasons could also add to the long-term viability and productivity of horse conch populations [49], although MPAs can be ineffective if they merely displace harvest pressure, temporally or spatially [50].

The way marine life landings data are collected could also be improved. Landings data for marine life trade are recorded only in “units,” individual specimens harvested live without data on weight or size (e.g., small juvenile vs large adult). A system that allows more specific data on sizes of harvested individuals is critical, however, because the risk to the population of removing hundreds of newly hatched conch is obviously different than the risks of removing hundreds of 400+ mm linear shell length females.

Some of the removed conch end up in the curio trade instead of the aquarium market. Web searches quickly return many dealers ranging from large commercial wholesale shops selling bulk quantities of shells on their own websites to individuals selling single shells on diverse platforms like Craigslist, eBay, and Etsy. Asking prices range from pennies per shell for newly hatched juveniles up to $500 for shells of 380 to 440 mm linear length https://www.ebay.com/itm/144325161665?hash=item219a7332c1:g:weIAAOSwO4xhPLJq, accessed 2/6/22). Shells larger than 450 mm, while likely available, are rare and would carry very high asking prices. The temptation for collectors or persons selling shells to harvest an exceptionally large horse conch is extreme. Such harvest would be legal under existing rules provided the person possessed a recreational fishing license or a commercial products license. Furthermore, because there are no state or federal regulations in Florida limiting the take of empty seashells, enforcement of any illegal take by an unlicensed harvester would require evidence the empty shells had been shucked or harvested live. Inclusion of the horse conch to the list of protected Marine Life would limit the recreational harvest of live specimens to five per person unless species-specific quantities were specified. There is currently no limit for commercial harvest of horse conch.

## Conclusions

The Florida Horse Conch has faced over a century of intense exploitation by shell collectors and commercial harvest, with independent lines of evidence suggesting that the species has long been in decline [6,10–12]. Several aspects of the horse conch’s biology make it particularly susceptible to population collapse, including its extremely large size and bright red foot, which make it easily visible to collectors, and its preference for shallow coastal habitats with easy access. To this list, we add the strong possibility that this species has a lifespan that is surprisingly short given its extreme large size and a predicted late age at first reproduction. Based on evidence for a decline in mean size of horse conchs since the 1960s [12], many females are likely harvested before they can spawn.

This study joins others [51,52] in highlighting how life history information necessary for managing species of threatened or declining mollusks can be obtained through stable isotope sclerochronology. This technique can be applied to specimens in historical museum collections, as we have done here, allowing better understanding of species’ maximum growth potential prior to modern human impacts and doing so without the need for sacrificing animals of already scarce species. The technique works on mollusk shells but also many other types of animals that have accretionary growth of mineralized hardparts, such as coral skeletons and fish vertebrae. Our study also demonstrates that isotope profiles of subtropical mollusks record sufficient seasonal variation to delineate time and measure growth rates and age. Most importantly, stable isotope sclerochronology of museum specimens can help circumvent a Catch-22 scenario playing out in marine management, where species in need of protection are now so rare that field research to establish intrinsic risk of extinction based on life history traits is no longer feasible, yet such information may be the only way to trigger management interventions. In the case of *T*. *giganteus*, the largest, oldest individuals have not been recorded in any modern surveys, which means application of stable isotope sclerochronology to shells in historic and prehistoric collections [e.g. 52] is the only way to study the maximum growth potential of the species. This information bottleneck will only get worse and the need for alternative research approaches more dire as species in need of protection become increasingly uncommon.

## Supporting information

S1 Fig Trace element/Ca ratios of shell lip cross sections. Mg/Ca (blue) and Sr/Ca (purple) ratios of shell lip cross sections. Black line represents oxygen isotope data from the corresponding area of shell.
(TIF)

S2 Fig Isotope sclerochronology profiles for juvenile *T*. *giganteus* shells. Oxygen and carbon isotope sclerochronology profiles for two juvenile shells, Tg-D and Tg-E (both BMSM 25347), from Sanibel Island, Florida.
(TIF)

S1 Appendix Isotope and shell measurement data. (XLSX)
