# Supplementary figures and images for: Age and growth of one of the world’s largest carnivorous gastropods, the Florida Horse Conch, Triplofusus giganteus (Kiener, 1840), a target of unregulated, intense harvest
Source: PLoS One. 2022 Apr 6;17(4):e0265095. doi: 10.1371/journal.pone.0265095 (PMC8985988; doi:10.1371/journal.pone.0265095)

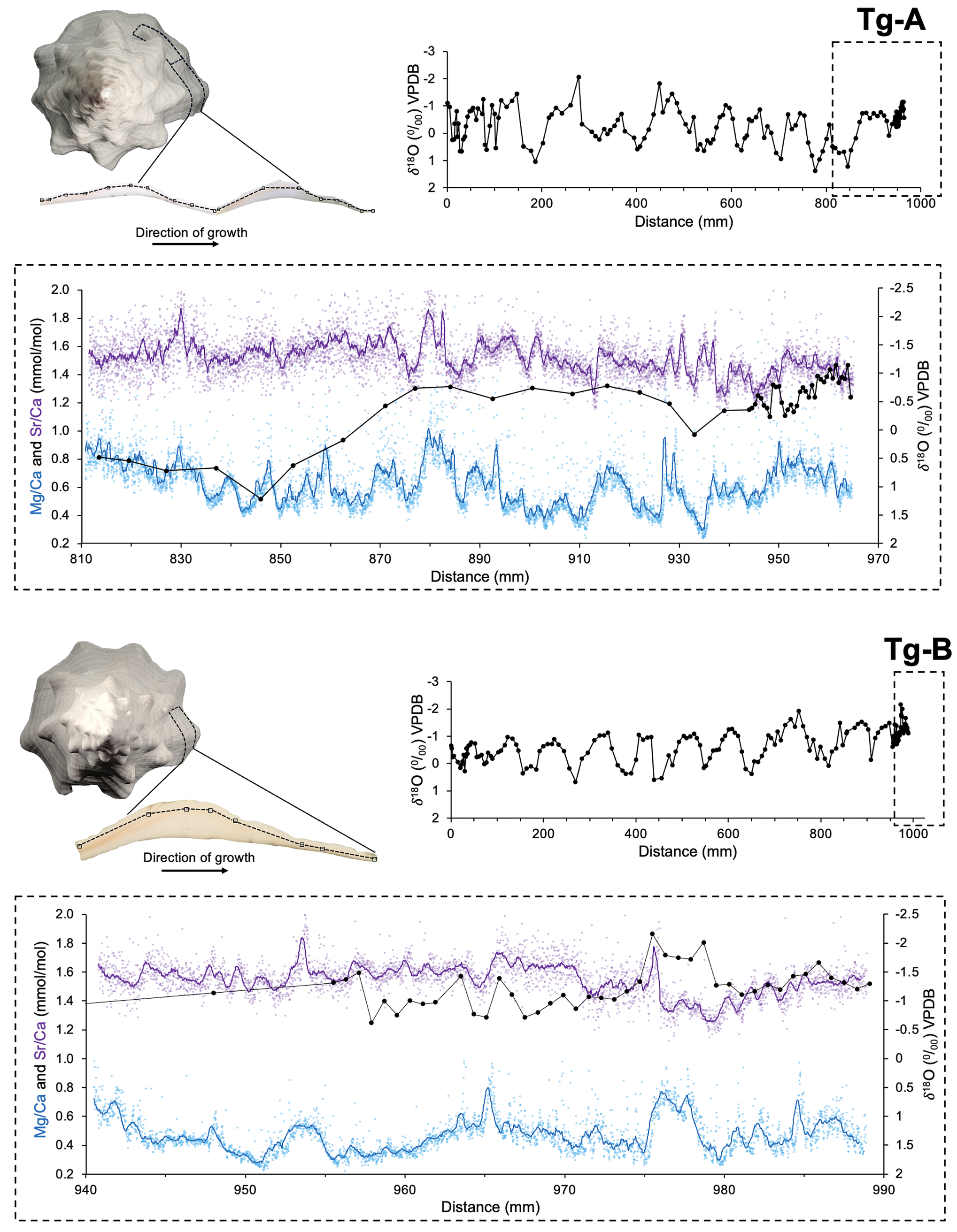

Supplement: S1 Fig — Mg/Ca (blue) and Sr/Ca (purple) ratios of shell lip cross sections. Black line represents oxygen isotope data from the corresponding area of shell. (TIF) [file pone.0265095.s001.tif]

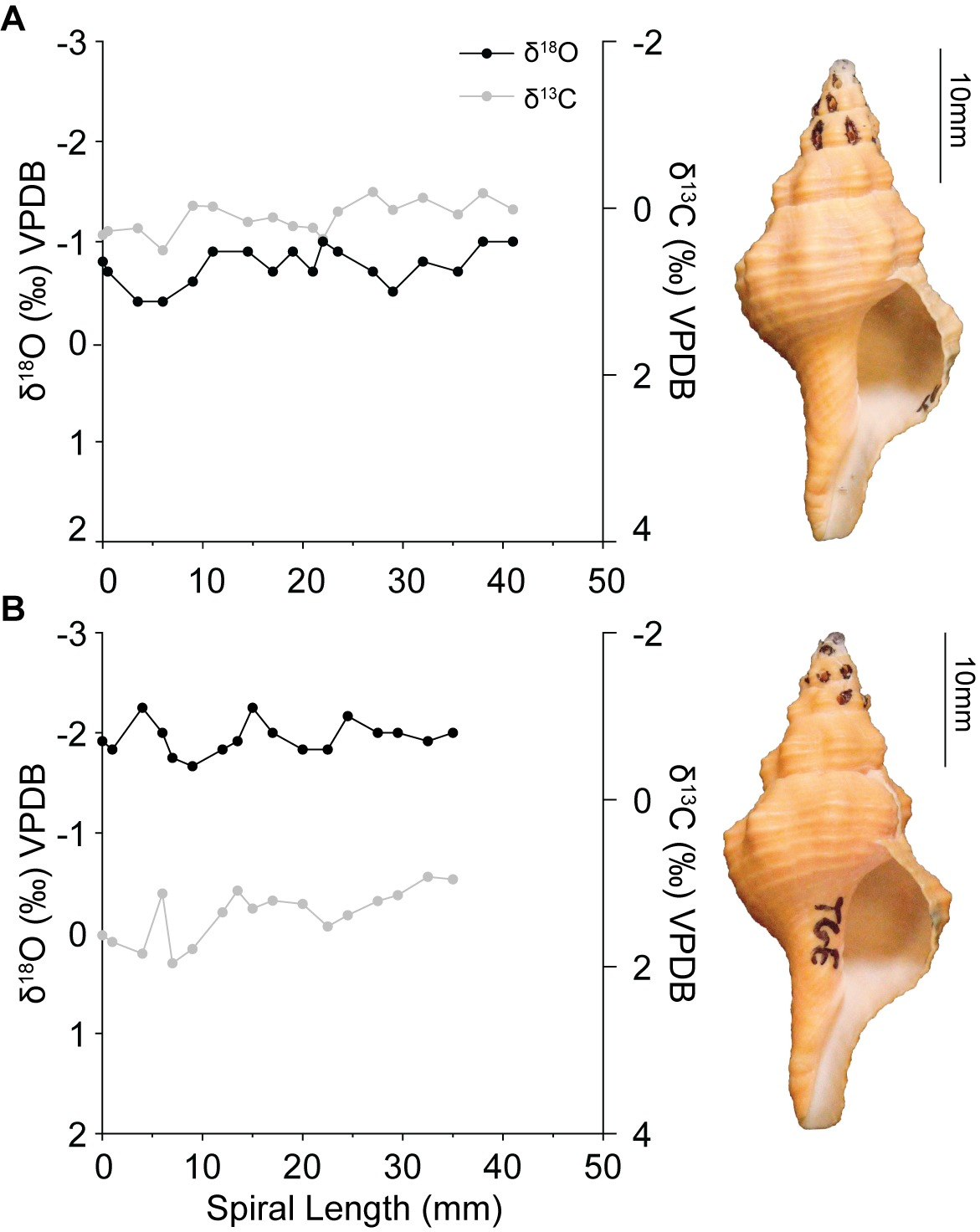

Supplement: S2 Fig — Oxygen and carbon isotope sclerochronology profiles for two juvenile shells, Tg-D and Tg-E (both BMSM 25347), from Sanibel Island, Florida. (TIF) [file pone.0265095.s002.tif]
